# Analysis of Short-Term and Stable DNA Damage in Patients with Differentiated Thyroid Cancer Treated with ^131^I in Hypothyroidism or with Recombinant Human Thyroid-Stimulating Hormone for Remnant Ablation

**DOI:** 10.2967/jnumed.121.263442

**Published:** 2022-10

**Authors:** Alberto Signore, Giuseppe Campagna, Jessica Marinaccio, Marco de Vitis, Chiara Lauri, Francesco Berardinelli, Anna Tofani, Marco Chianelli, Marina Borro, Giovanna Gentile, Maurizio Simmaco, Francesco Colombini, Anna Giovanetti, Antonella Sgura

**Affiliations:** 1 Nuclear Medicine Unit, Department of Medical-Surgical Sciences and of Translational Medicine, Faculty of Medicine and Psychology, University of Rome “Sapienza,” Rome, Italy;; 2 Department of Science, University of Rome “Roma Tre,” Rome, Italy;; 3 Unit of Endocrinology, Regina Apostolorum Hospital, Rome, Italy;; 4 Department of Neurosciences, Mental Health, and Sensory Organs, Faculty of Medicine and Psychology, University of Rome “Sapienza,” Rome, Italy;; 5 Diacron International SRL, Grosseto, Italy; and; 6 ENEA, Division of Health Protection Technologies, Casaccia Research Centre, Rome, Italy

**Keywords:** ^131^I, radiation-induced genetic damage, hypothyroidism, rhTSH, gene polymorphism, free oxygen radicals

## Abstract

It is well known that ionizing radiation can induce genetic damage and that oxidative stress is a major factor inducing it. Our aim was to investigate whether thyroid remnant ablation with low activities of ^131^I (1,850 MBq) is associated with DNA damage by evaluating the CometAssay, micronuclei, and chromosome aberrations with multicolor fluorescent in situ hybridization. **Methods:** We studied 62 patients prepared with recombinant human thyroid-stimulating hormone (rhTSH) or by thyroid hormone withdrawal. In both groups, we analyzed stable and unstable genetic alterations before ^131^I therapy and 1 wk and 3 mo after ^131^I administration. We also correlated the genetic damage with several variables, including the degree of radiation-induced oxidative stress, genetic polymorphisms of enzymes involved in DNA repair, and antioxidative stress. **Results:** We found a comparable amount of DNA breaks evaluated by CometAssay and micronuclei testing in both groups of patients at different time points, but there was a significant increase in stable chromosome aberrations evaluated by multicolor fluorescent in situ hybridization (breaks and translocations) in patients prepared with thyroid hormone withdrawal. Overall, high chromosome damage was associated with higher retained body radioactivity and unfavorable gene polymorphism. A high level of free oxygen radicals and a low level of antioxidants were found in all patients at any time point. In particular, patients prepared with thyroid hormone withdrawal, at 3 mo, had significantly higher levels of free oxygen radicals than those prepared with rhTSH. **Conclusion:** An increase in stable chromosome aberrations with respect to baseline is detectable after administration of low doses of ^131^I in patients prepared with thyroid hormone withdrawal but not in patients prepared with rhTSH. The clinical significance of these chromosomal alterations remains to be determined.

## Visual Abstract

**Figure:**
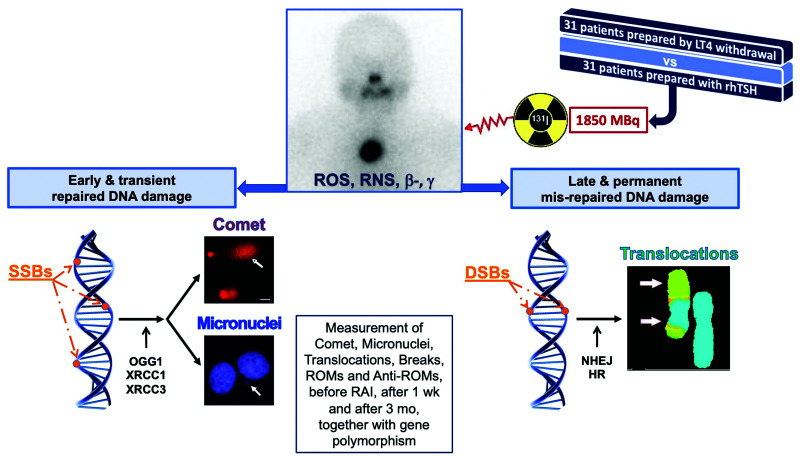

In clinical practice, ^131^I is used for thyroid remnant ablation in patients who undergo thyroidectomy for differentiated thyroid carcinoma. Radionuclide therapy has been reported to induce harmful effects on cells and tissues (*1*–*7*). Indeed, several reports have shown chromosomal damage induced by ^131^I, although only chromosomes 1, 2, 4, 8, and 10 have been analyzed (*8*–*12*). Despite this controversial issue (*3**,**5**,**13*–*19*), a significant reduction in ablative treatments has been observed in the last decade. Therefore, it is important to better elucidate the possible presence of stable genetic damage and of radioinduced oxidative stress after treatment with ^131^I. Furthermore, gene polymorphisms that alter the repair of DNA damage should also be investigated (*20*).

Recombinant human thyroid-stimulating hormone (rhTSH) has effectively been used for exogenous stimulation before ^131^I ablation therapy, although it is not yet widely used for preparation of patients receiving high therapeutic amounts of radioiodine (*5*). The main aim of this study was to investigate whether the relatively low administered activities of ^131^I for thyroid remnant ablation are associated with some stable chromosome damage. Secondary aims were to analyze the level of baseline (i.e., before remnant ablation) genetic damage and oxidative stress in patients with differentiated thyroid carcinoma; to evaluate the role of different DNA repair and antioxidative genes in the occurrence of genetic damage and oxidative stress by analyzing genetic polymorphisms in patients; and to evaluate whether the yield of damage is comparable in patients prepared by rhTSH or by hypothyroidism.

## MATERIALS AND METHODS

### Patients

A group of 62 patients to be treated with 1,850 MBq (50 mCi) of ^131^I were randomly assigned to 2 cohorts: 31 patients in hypothyroidism (HYPO group) (40-d suspension of levothyroxine and replacement with triiodothyronine for the first 25 d) and 31 patients in euthyroidism injected with rhTSH (rhTSH group) (1 mg 2 d before ^131^I and 1 mg 1 d before ^131^I).

Patients were matched for age, sex, pathologies, and lifestyle habits; were nonsmokers; and were being administered no drugs. Patients with other primary tumors or previously treated with radiotherapy or receiving drugs with an effect on oxidative status or on the immune system were excluded. All patients were on a low-iodine diet for 10 d before therapy. The study was approved by local Ethics Committee (approvals 736/2014 and 241 SA_2017), and all subjects gave written informed consent. Patient recruitment lasted 15 mo; the study was completed in 2 y.

The radiation exposure rate was measured at a 1-m distance at the time of ^131^I administration (baseline), after 24 h, and after 48 h, as an indirect measurement of residual body activity. Patients with less than 20 μSv/h at 48 h were discharged from the hospital. Those with more than 20 μSv/h were counted again after 72 h.

### Sampling

In all patient cohorts, 10 mL of blood were withdrawn in lithium heparin.

Before treatment with ^131^I in euthyroidism (when sampling for therapeutic purposes was scheduled), blood was immediately processed for micronuclei, translocations, DNA breaks (CometAssay; Bio-Techne Corp.), thyroglobulin levels, creatinine, glomerular filtration rate estimated with the Chronic Kidney Disease Epidemiology Collaboration formula (epi-GFR) (*21*), thyroid stimulating hormone (TSH), plasma reactive oxygen metabolite-derived compounds (d-ROMs), plasma anti–reactive oxygen metabolite potential (anti-ROMs), and single-nucleotide polymorphisms (SNPs).

One week after ^131^I treatment (when sampling for therapeutic purposes was scheduled), blood was immediately processed for micronuclei, CometAssay, d-ROMs, and anti-ROMs.

Three months after ^131^I treatment (when sampling for follow-up purposes was scheduled), blood was processed for micronuclei, translocations, CometAssay, thyroglobulin, TSH, d-ROMs, and anti-ROMs.

### Cell Culture Conditions

Half-milliliter blood samples from patients were diluted with 4.5 mL of complete medium in a culture flask and incubated at 37°C in a humidified atmosphere with 5% CO_2_. The culture medium was RPMI 1640 (Euroclone) supplemented with 20% heat-inactivated fetal bovine serum (Euroclone), penicillin (10,000 units/mL) and streptomycin (10 mg/mL) (Biologic Industries), and 1% l-glutamine (Euroclone). T lymphocytes were stimulated to divide for 72 h using 2% phytohemagglutinin (Gibco) in the culture medium.

### Micronucleus Assay

To obtain binucleated cells, cytochalasin B (6 μg/mL) (Sigma Aldrich) was added to the culture medium 24 h before harvesting, as previously described (*22*). Briefly, cells were pelleted by centrifugation (8 min at 12,000 rpm), resuspended in 0.075 M KCl, and incubated for 2 min at 37°C. The suspension was fixed 3 times in freshly prepared modified Carnoy solution (5:1 v/v methanol/acetic acid). Binucleated cells were dropped onto slides, air-dried, and counterstained with 4,6-diamidino-2 phenylindole (Sigma Aldrich) in Vectashield antifade (Vector Laboratories). Micronuclei were identified according to the following criteria: the micronuclei were in cytoplasm and had a diameter of less than a third of the whole nucleus; they were circular or oval, and their staining and refractivity were in accordance with that of the whole nucleus; and their structures were similar to those of the whole nucleus, with complete separation and no other nearby fragments or impurities. Images were captured with the Metacyte module of Metafer automated capture software (MetaSystems) at ×40 magnification using an Axio Imager Z1 microscope (Zeiss) equipped with a Cool Cube 1 (charge-coupled device) camera (MetaSystems). At least 1,000 binucleated cells for each patient were analyzed under each experimental condition.

### CometAssay

The CometAssay technique was used to evaluate the frequency of double-strand breaks and single-strand breaks induced by ^131^I.

The alkaline CometAssay was performed as described by Giovanetti et al. (*23*). Twenty microliters of whole blood were gently resuspended in 180 μL of 0.7% low-melting-point agarose in phosphate-buffered saline (calcium- and magnesium-free) at 38°C and immediately pipetted onto a warm frosted glass microscope slide precoated with a layer of 1% normal-melting-point agarose in phosphate-buffered saline. Coverslips were applied, and the slides were set at 4°C to solidify the agarose. The coverslips were then removed, and the slides were incubated in a lysis solution (2.5 M NaCl, 10 mM Tris-HCl, 100 mM Na_2_ ethylenediaminetetraacetic acid, NaOH to pH 10, 1% Triton X-100 (Thermo Fisher Scientific), and 10% dimethyl sulfoxide) for 45 min. After this step, all the operations were performed at 4°C under dim light. After lysis, the slides were rinsed for 10 min with electrophoresis buffer (1 mM Na_2_-ethylenediaminetetraacetic acid, 300 mM NaOH, pH 13) and placed for 20 min onto a horizontal electrophoresis unit containing the same electrophoresis buffer to allow DNA unwinding. Electrophoresis was conducted with the Sub-Cell GT System (15 Å to ∼25 cm) equipped with Power Pack 300 (Bio-Rad Laboratories Inc.) for 15 min (25 V, 300 mA). Subsequently, the slides were gently washed in neutralization buffer solution for 5 min (0.4 M Tris-HCl, pH 7.5), dehydrated with an ethanol series (70%, 85%, and 100%), dried at room temperature, and stored. When not otherwise indicated, all chemicals were purchased from Sigma Aldrich.

For microscopy analysis, the slides were stained with ethidium bromide (10 μg/mL) immediately before being analyzed at ×400 magnification by a fluorescent Axiolab Zeiss microscope (Carl Zeiss AG).

The slides were analyzed using a fluorescence microscope (Leica) equipped with a camera. On each slide, coded and (with masking) scored, 200 comets were acquired using the I.A.S. software automatic image analysis system (Delta Sistemi).

### Collection of Chromosome Spreads and Multicolor Fluorescent In Situ Hybridization (M-FISH)

The M-FISH analysis was used to quantify stable genomic damage due to ^131^I. We analyzed the following in particular: exchanges (both simple exchanges, caused by 2 breaks on 2 different chromosomes [reciprocal and nonreciprocal], and complex exchanges, due to 3 or more breaks on 2 or more chromosomes); acentric fragments; and total breaks (as the total number of breakpoints involved in simple and complex exchanges and acentric fragment observed).

Chromosome spreads were obtained after 3 h of incubation in 5 × 10^6^ M colchicine (Sigma Aldrich). Metaphase spreads were prepared following standard cytogenetic procedures, consisting of treatment with a hypotonic solution (0.075 M KCl) for 20 min at 37°C followed by fixation in freshly prepared Carnoy solution (3:1 v/v methanol/acetic acid).

Fixed cells were dropped onto glass slides and hybridized with the 24XCyte Human M-FISH Probe Kit (MetaSystems), as previously reported (*24**,**25*). Briefly, the slides were denatured in 0.07N NaOH and then rinsed in a graded ethanol series. Meanwhile, the probe mix was denatured using an MJ mini personal thermal cycler (Bio-Rad Laboratories) with the following program: 5 min at 75°C, 30 s at 10°C, and 30 min at 37°C. Probes were added to the slides, and a coverslip was added and sealed using rubber cement. Samples were then hybridized in a humidified chamber at 37°C for 48 h, washed in saline–sodium citrate buffer for 5 min at 75°C, and counterstained with 4,6-diamidino-2 phenylindole (Sigma Aldrich) in Vectashield antifade (Vector Laboratories). Finally, images were captured with the M-search module of Metafer software (MetaSystems) at ×63 magnification using an Axio Imager Z1 microscope (Zeiss) equipped with a Cool Cube 1 camera (MetaSystems). At least 100 metaphases were analyzed for each patient under each experimental condition. The karyotyping and cytogenetic analyses of each single chromosome were performed using the ISIS software (MetaSystems).

### Genotyping (SNPs)

Genomic DNA was isolated from blood samples using the X-tractor Gene system (Corbett Life Science). Reference sequences for each gene were obtained from NCBI GenBank database. The sequences of selected primers are reported in Supplemental Tables 1 and 2 (supplemental materials are available at http://jnm.snmjournals.org). Locus-specific PCR and extension primers were designed by Genotyping Tools and the MassArray Assay Design 4.0 software (Sequenom Inc.).

The SNPs analyzed were XRCC1 G28152A (rs25487), XRCC3 A4541G (rs1799794), XRCC3 C18067T (rs861539), and RAD51 G315C (rs1801320) for enzymes used to repair single-strand DNA breaks (DNA1 package) and CAT C-262T (rs1001179), OGG1 Ser326Cys (rs1052133), NOS3 Glu298Asp (rs1799983), PON1 A575G (rs662), PON1 C-108T (rs705379), and MPO G-463A (rs2333227) for enzymes used to scavenger activity of free oxygen (DNA2 package).

Genotyping of XRCC1 G28152A (rs25487), XRCC3 A4541G (rs1799794), XRCC3 C18067T (rs861539), and RAD51 G315C (rs1801320) was performed by pyrosequencing technology, using the PyroMark Q48 Autoprep system (Qiagen) according to manufacturer directions. Both the amplification and the sequencing primers were obtained by the PSQ Assay Design software (Qiagen).

The region covering the SNPs of interest was amplified by polymerase chain reaction (PCR). The PCR conditions were 95°C for 3 min, 40 cycles with denaturation at 95°C for 30 s, annealing at 56°C for 30 s, elongation at 72°C for 30 s, and a final extension step at 72°C for 5 min. PCR was performed in a final volume of 25 μL, containing 70 ng of genomic DNA, 10 pmol of each primer, 0.2 mM dNTPs, PCR buffer, 1 U of Taq DNA polymerase (Takara Bio Inc.), and 1 mM MgCl_2_ for XRCC1 G28152A, XRCC3 C18067T, and RAD51 G315C, whereas 1.5 mM MgCl_2_ was used for XRCC3 A4541G amplification (*26*).

The other SNPs were genotyped by the Sequenom MassArray iPLEX platform (Sequenom). Twenty nanograms of genomic DNA were standardized for genotyping of each sample. According to the manufacturer’s instructions, the DNA samples were amplified by a multiplex PCR and treated with shrimp alkaline phosphate. The PCR products were then used for locus-specific single-base extension reaction. The resulting products were desalted and transferred to a 96-SpectroCHIP array. The alleles were discriminated by matrix-assisted laser desorption ionization time-of-flight mass spectrometry. Data were processed and analyzed by Sequenom MassArray TYPER 4.0 software.

For each heterozygote mutation, we assigned a score of 0.5, and for each homozygote mutation, we assigned a score of 1. The total mutation score for DNA-1 and DNA-2 enzyme packages was calculated for each patient.

### Oxidative Stress and Antioxidant Capacity

d-ROMs and anti-ROMs were measured with Diacron kits (Diacron International). The d-ROMs test measures the oxidant ability of a plasma sample toward a particular substance (modified aromatic amine) used as an indicator (chromogen). The change in absorbance per unit time (calculated on the basis of a serum with known title) is expressed in conventional units (CARR U). The reference range is less than 300 CARR U (*27*).

The anti-ROMs test measures the antioxidant capacity of plasma, expressed as iron-reducing activity. The method has been engineered to have 2 phases: the first phase (first minute) provides the value of the so-called fast antioxidants (anti-ROMsF) (i.e., those that act quickly, such as vitamin C or vitamin E), and the second phase provides the value of the so-called slow antioxidants (anti-ROMsS) (such as thiol groups, sulfhydryl groups [-SH], uric acid, or polyphenols). The test reference values in a healthy population are more than 200 μEq/L for fast antioxidants and more than 1,000 μEq/L for slow antioxidants (*28*).

### Statistical Analysis

Sample size was calculated on the basis of the results of M-FISH translocations observed in an unpublished previous pilot study on 10 patients. We hypothesized a clinically meaningful difference of 0.8 for μ_HYPO_ − μ_rhTSH_, with an SD of 1.1 and setting α to 0.05 with 80% power. The calculated total sample size was 62 patients, namely 31 per group.

Continuous variables are presented as mean ± SD when data were normally distributed or as median and 95% CI otherwise. Categoric variables are expressed as absolute frequencies and percentages.

The association of group (HYPO vs. rhTSH) with sex, pT1, pT3, and papillary and follicular histologic type was evaluated by the χ^2^ test, whereas the association with pT2, pT4, and N1 was evaluated by the Fisher exact test because the expected frequencies were less than 5. The Shapiro–Wilk test was used to test the normality of the continuous variables and of the residuals, whereas homoscedasticity was verified by checking the studentized residuals. For continuous variables, differences between groups (HYPO vs. rhTSH) were compared by the Student *t* or Mann–Whitney test. A generalized linear mixed model for repeated measures with a gaussian distribution and an identity link was used to verify differences in d-ROMs, anti-ROMsS, and anti-ROMsF at baseline, 1 wk, and 3 mo in the HYPO group versus the rhTSH group. The Tukey method was used to correct the *P* values for multiple comparisons.

To understand which variables may influence the break or total exchange at the 3-mo time point in HYPO and rhTSH patients, we used a generalized linear mixed model with a negative binomial function and a logarithmic link, with independent variables consisting of age; sex; breaks or total exchanges at baseline; radiation exposure rate at 48 h; anti-ROMsF, anti-ROMsS, and d-ROMs at baseline and 3 mo; and DNA-1, DNA-2, and epi-GFR at baseline.

To evaluate differences in micronuclei and CometAssay between the 3 temporal points (baseline, 1 wk, and 3 mo) relative to HYPO versus rhTSH, we applied a generalized linear mixed model for repeated measures with a negative binomial/gaussian distribution and a logarithmic/identity link (respectively), and the Tukey method was used to correct for multiple comparisons.

Finally, a generalized linear mixed model with a negative binomial distribution and a logarithmic link was used to assess differences in breaks and total exchanges between baseline and 1 wk in the HYPO group versus the rhTSH group. The Tukey method was used to correct for multiple comparisons.

A *P* value of less than 0.05 was considered statistically significant. Data were analyzed by SAS, version 9.4 (SAS Institute Inc.).

## RESULTS

All patients completed the 1-y follow-up. None dropped out, but 2 patients did not consent to analysis of DNA polymorphisms, and in 3 patients of the rhTSH group we did not measure d-ROMs and anti-ROMs. The mean age of the 2 groups did not significantly differ (Table 1), but a significant difference was seen in the levels of TSH, thyroglobulin at the time of ^131^I, creatinine, and epi-GFR.

**TABLE 1.** Differences in Clinical and Biochemical Variables Between HYPO and rhTSH Groups

Variable	HYPO	rhTSH	*P*
Age (y)	47.64 ± 11.18 (43.54–51.75)	48.64 ± 11.53 (44.42–52.87)	0.73
Sex			0.32
Male	4 (12.90%)	7 (22.58%)	
Female	27 (87.10%)	24 (77.42%)	
pT1	18 (58.06%)	20 (64.52%)	0.60
pT2	5 (16.13%)	1 (3.23%)	0.19
pT3	7 (22.58%)	10 (32.26%)	0.39
pT4	1 (3.23%)	0 (0.00%)	1.00
Papillary histologic type	20 (64.52%)	19 (61.29%)	0.79
Follicular histologic type	11 (35.48%)	12 (38.71%)	0.79
N1	2 (6.45%)	4 (12.90%)	0.67
TSH at ^131^I (μU/mL)	87.37 (76.62–99.72)	112.80 (98.84–135.80)	0.01
Thyroglobulin at ^131^I (ng/mL)	0.79 (0.25–2.77)	0.25 (0.20–0.34)	0.008
Thyroglobulin at 3 mo (ng/mL)	0.10 (0.10–0.10)	0.10 (0.10–0.10)	0.53
Exposure rate at 24 h (mSv/h)	33 (28–37)	24 (20–28)	<0.0001
Exposure rate at 48 h (mSv/h)	16 (12–18)	9 (8–9)	<0.0001
Creatinine (mg/mL)	0.98 (0.85–1.05)	0.76 (0.72–0.80)	<0.0001
Epi-GFR (mL/min)	74.77 ± 17.50 (68.36–81.19)	97.61 ± 12.50 (93.03–102.20)	<0.0001

Qualitative data are number and percentage; continuous data are mean ± SD or median and 95% CI.

Administration of rhTSH was well tolerated. All patients had efficient ablation as evaluated by unstimulated thyroglobulin levels at 3 mo after therapy and by rhTSH-stimulated thyroglobulin levels at the 12-mo follow-up.

**FIGURE 1.**
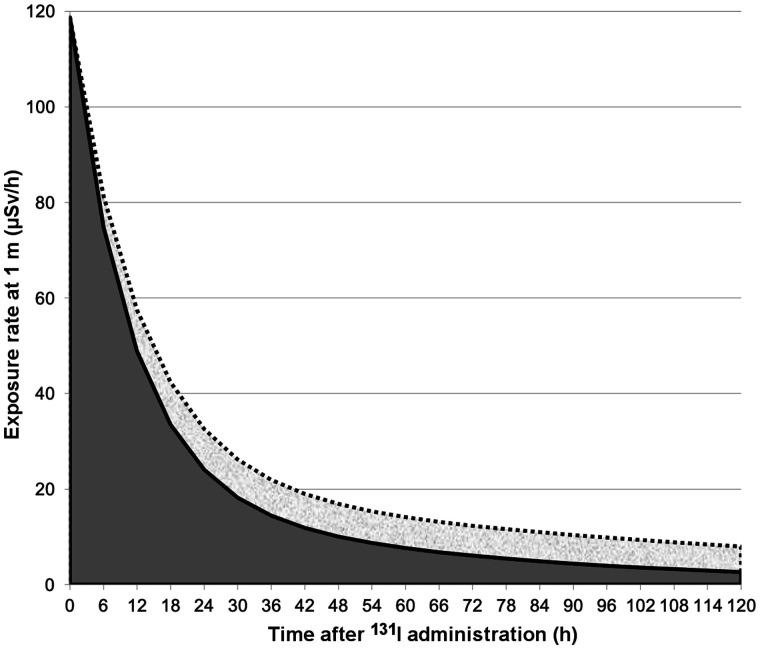
Results of fitted radiation exposure rate in patients, up to 120 h, measured at 1-m distance. Dotted line represents HYPO patients; solid line represents rhTSH patients. In HYPO patients, there is increased retained body activity with respect to rhTSH patients, with 27% higher exposure rate, calculated comparing 2 areas under curves.

The radiation exposure rate at 1 m was higher in HYPO patients on days 1 and 2 than in rhTSH patients (median, 36 vs. 24 μSv/h on day 1 and 16 vs. 9 μSv/h on day 2; both *P* < 0.0001; Table 1). Most patients were dismissed on day 2, but 4 patients from the HYPO group were dismissed on day 3 and 11 patients from the rhTSH group were dismissed on day 1, confirming the lower residual body activity in rhTSH patients than in HYPO patients. The calculated area under the curve for the exposure rate through 120 h was 27% lower in rhTSH patients than in HYPO patients, reflecting lower residual body activity (Fig. 1).

### Micronucleus and CometAssay

**FIGURE 2.**
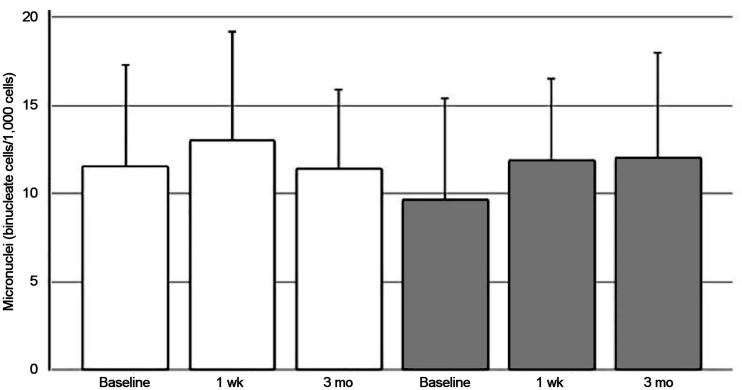
Results of micronucleus measurement (white bars, HYPO; gray bars, rhTSH). No differences exist between or within groups. Tukey method was used to correct for multiple comparisons.

Micronucleus values did not differ between the HYPO and rhTSH group or between the same group at 1 wk and 3 mo after treatment (Fig. 2).

**FIGURE 3.**
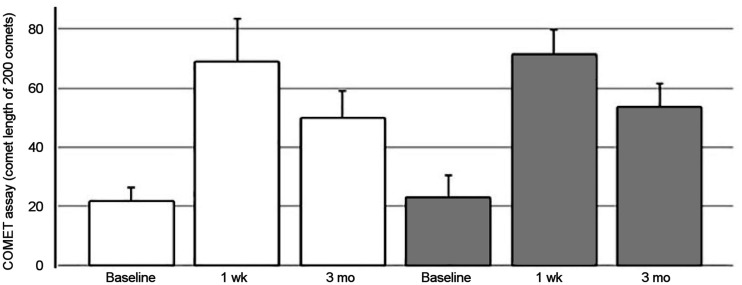
Results of CometAssay (white bars, HYPO; gray bars, rhTSH). Significant differences were found between baseline and 1 wk, between 1 wk and 3 mo, and between baseline and 3 mo in both groups (all *P* < 0.0001). Tukey method was used to correct for multiple comparisons.

The CometAssay showed no differences between the HYPO and rhTSH groups. However, all patients showed a statistically significant increase in damage at 1 wk (*P* < 0.0001), followed by a reduction at 3 mo (*P* < 0.0001), although not yet reaching the basal values (Fig. 3).

### M-FISH

**FIGURE 4.**
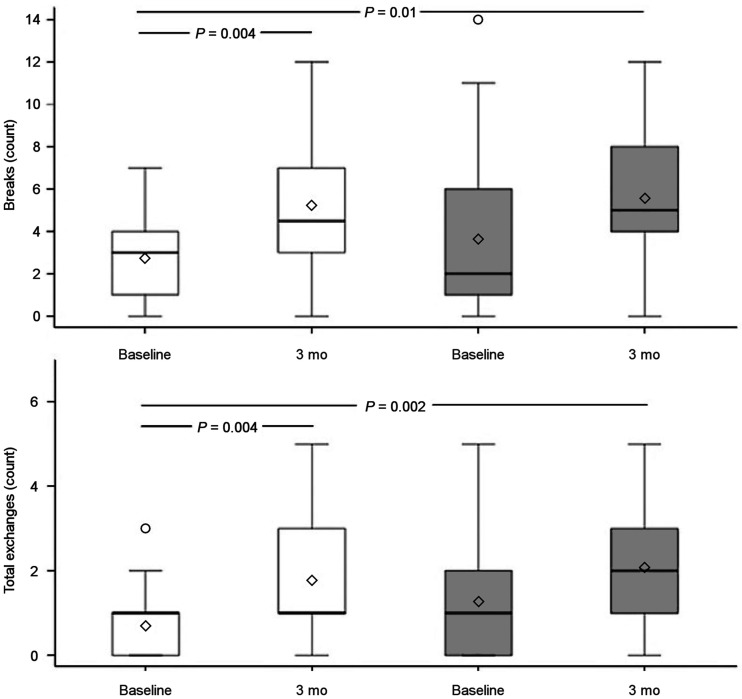
Stable chromosome damage (total exchanges and chromosome breaks) in patients before and 3 mo after ^131^I therapy (white bars, HYPO; gray bars, rhTSH). Tukey method was used to correct for multiple comparisons.

Results are reported in Figure 4 and Table 2. In particular, breaks and total exchanges in patients belonging to the HYPO group significantly increased from the basal sample to the 3-mo sample (in both, *P* = 0.004). By contrast, in patients belonging to the rhTSH group, no significant increase in breaks or total exchanges was found between the basal sample and the 3-mo sample. Overall, 19 of 32 patients from the rhTSH group had no increase or even a reduction in the number of chromosome breaks, as compared with 8 of 32 patients from the HYPO group. If we consider the total exchanges, 23 of 32 patients from the rhTSH group had no increase or even a reduction in the number of chromosome exchanges, as compared with 11 of 32 patients from the HYPO group.

**TABLE 2.** Parameter Estimates by Generalized Linear Mixed Model of Chromosome Breaks and Exchanges at 3 Months in HYPO and rhTSH Groups

Variable	Mean ± SE	95% CI	Exp (mean)	% chance	*P*
Chromosome breaks at 3 mo in HYPO patients*					
Anti-ROMsS at 1 wk	−0.003 ± 0.001	−0.005 to −0.00005	1.00	−0.3	0.046
Exposure rate at 48 h	0.06 ± 0.02	0.01 to 0.10	1.06	6.2	0.016
Chromosome exchanges at 3 mo in HYPO patients^†^					
Anti-ROMsS at 1 wk	−0.009 ± 0.003	−0.02 to −0.002	0.99	−0.9	0.015
DNA-1	0.82 ± 0.34	0.10 to 1.54	2.28	127.5	0.027
Exposure rate at 48 h	0.06 ± 0.03	0.002 to 0.12	1.06	6.2	0.046
Chromosome breaks at 3 mo in rhTSH patients^‡^					
DNA-2	0.67 ± 0.25	0.03 to 1.31	1.95	95.4	0.04

* Covariate: age, sex, breaks at baseline, d-ROMs at baseline and 1 wk, anti-ROMsF at baseline and 1 wk, anti-ROMsS at baseline, DNA-1, DNA-2, and epi-GFR.

^†^Covariate: age, sex, exchanges at baseline, d-ROMs at baseline and 1 wk, anti-ROMsF at baseline and 1 wk, anti-ROMsS at baseline, DNA-2, and epi-GFR.

^‡^Covariate: age, sex, breaks at baseline, d-ROMs at baseline and 1 wk, anti-ROMsF at baseline and 1 wk, anti-ROMsS at baseline and 1 wk, DNA-1, dosimetry at 48 h, and epi-GFR.

Exp (mean) = exponential of the mean.

Table 2 shows the results of the generalized linear mixed model of breaks and total exchanges at 3 mo in HYPO and rhTSH patients. In the HYPO group, anti-ROMs at 1 wk were negatively associated with breaks at 3 mo (with a reduction of 0.3%; *P* = 0.046), whereas the exposure rate at 48 h was positively associated with the response variable, with an increase of 6.2% (*P* = 0.016). Total chromosome exchanges at 3 mo in the HYPO group were negatively associated with anti-ROMs at 1 wk, with a reduction of 0.9% (*P* = 0.015), and were positively associated with DNA-1, with an increase of 127.5% (*P* = 0.027). The exposure rate at 48 h was positively associated with the response variable, with an increase of 0.2% (*P* = 0.046). Finally, in the rhTSH group, only exposure rate at 48 h was positively associated with breaks at 3 mo in the rhTSH group, with an increase of 95.4% (*P* = 0.04).

No association was observed between total exchanges at 3 mo in the rhTSH group and the analyzed covariates.

### Genotyping (SNPs)

In the HYPO patient group, 11 patients had a total DNA score (DNA-1 + DNA-2) of 3 or higher, but in the rhTSH patient group, 17 patients had a total DNA mutation score of 3 or higher, indicating a higher frequency of mutations in rhTSH patients. These polymorphisms were observed predominantly in genes of the DNA-2 package (for the antioxidative stress enzymes) and, indeed, positively influenced the number of chromosome breaks at 3 mo in rhTSH patients.

Nevertheless, in the HYPO subgroup with a DNA mutation score of at least 3, chromosome breaks measured by the M-FISH technique increased from 2.3 ± 2.2 (mean basal sample) to 5.5 ± 2.8 (3-mo sample) (*P* = 0.01), as compared with rhTSH patients (with DNA mutation score ≥ 3), in whom the frequency of chromosome exchange breaks increased from 6.6 ± 8.1 (basal sample) to 8.5 ± 8.6 (3-mo sample) (*P* = not statistically significant).

Chromosome total exchanges in these HYPO patients also significantly increased from 1.0 ± 1.1 to 2.1 ± 1.1 (basal vs. 3-mo sample) (*P* = 0.03), as compared with rhTSH patients, in whom the frequency of chromosome exchange breaks increased from 2.1 ± 3.0 to 3.3 ± 4.6 (basal vs. 3-mo sample) (*P* = not statistically significant).

### Oxidative Stress

Overall, most patients had high levels of d-ROMs and low levels of anti-ROMs at any time point, with no significant differences between HYPO patients and rhTSH patients at baseline and 1 wk but higher values in HYPO patients at 3 mo (*P* = 0.03) (Table 3). Only 3 HYPO patients and 4 rhTSH patients had normal d-ROM values at entry, highlighting the high level of stress induced by cancer, surgery, and the postsurgical period (including hypothyroidism in HYPO patients) or supraphysiologic thyroxine replacement.

**TABLE 3.** Differences in Longitudinal Data of d-ROMs and Anti-ROMsS/F Between HYPO and rhTSH Groups

	HYPO				rhTSH			
Variable	Baseline	1 wk	3 mo	*P*	Baseline	1 wk	3 mo	*P*
d-ROMs	399.75 ± 86.51 (368.02–431.48)	333.46 ± 71.07 (307.39–359.53)	402.93 ± 72.60* (375.82–430.04)	<0.0001	374.22 ± 75.56 (345.72–402.72)	355.20 ± 93.50 (319.93–390.47)	363.62 ± 66.84* (338.20–389.04)	0.29
Anti-ROMsF	209.15 ± 50.53 (190.61–227.68)	215.13 ± 47.25 (197.80–232.46)	212.13 ± 52.46 (192.89–231.37)	0.83	211.49 ± 35.75 (197.63–225.36	219.53 ± 36.46 (205.39–233.67)	202.81 ± 30.95 (191.46–214.16)	0.07
Anti-ROMsS	727.15 ± 121.06 (682.75–771.55)	731.30 ± 172.46 (668.04–794.56)	727.31 ± 134.85 (677.85–776.77)	0.99	681.79 ± 116.81 (636.50–727.09)	720.57 ± 96.55 (683.13–758.01)	665.52 ± 105.12 (626.96–704.08)	0.09

* HYPO vs. rhTSH: d-ROMs: *P* (3 mo vs. 3 mo) = 0.03.

Post hoc analysis: HYPO. d-ROMs: *P* (baseline vs. 1 wk) = 0.0001; *P* (1 wk vs. 3 mo) < 0.0001. Normal values are <300 CARR U for d-ROMs, >200 μEq/L for anti-ROMsF, and >1,000 μEq/L for anti-ROMsS. Data are mean ± SD and 95%CI.

In HYPO patients, we observed a reduction in oxidative stress at 1 wk, with a statistically significant decrease in d-ROMs versus baseline (*P* = 0.0001). However, at 3 mo, HYPO patients had a new significant increase in d-ROMs as compared with values at 1 wk (*P* < 0.0001). These differences were not observed in rhTSH patients.

As far as anti-ROMsF and anti-ROMsS are concerned, we did not observe significant differences between HYPO and rhTSH patients or significant modifications over time. However, most patients had anti-ROM values below the level of reference ranges at any time, presumably as a result of the high stress level in these patients.

## DISCUSSION

Several papers have been published on the potential genetic damage and on the increased risk of secondary cancer in patients treated with ^131^I, even if with low activities (*1*–*20*), although the causative association between ^131^I therapy and an increased risk of secondary cancers has not yet been definitively established. Indeed, no longitudinal study has been published indicating that an increase in chromosome breaks or translocations after ^131^I therapy is associated with an increased risk of malignancies. Nevertheless, there is a worldwide tendency to reduce ^131^I ablation in patients with a low or intermediate risk after total thyroidectomy for differentiated thyroid carcinoma.

Structural aberrations generated by DNA double-strand breaks can be classified as stable or unstable according to their ability to persist in cellular progeny. Unstable aberrations include deletions, dicentric chromosomes, ring chromosomes, and acentric or otherwise asymmetric rearrangements that are normally not tolerated (i.e., lethal) in dividing cells and are, therefore, not transmitted with subsequent cell divisions. Stable aberrations, on the other hand, are generally tolerated by the cells and transmitted to the following cellular generations. It is believed that stable and unstable aberrations are induced with the same frequency, but unstable aberrations seem to be less frequent precisely because they are lost at each cell division.

We investigated whether 1,850 MBq (50 mCi) of ^131^I for ablation therapy could cause stable genetic damage in patients who undergo surgery for differentiated thyroid carcinoma with low and intermediate risk. Moreover, we investigated whether patients prepared with thyroid hormone withdrawal or with rhTSH display similar levels of chromosome damage. We found transient unstable DNA damage in both groups and modest stable DNA damage only in HYPO patients.

Indeed, micronuclei are a sign of early unstable damage, and we expected normal levels at 1 wk and 3 mo after therapy. The trend of DNA damage measured by CometAssay after 1 wk and 3 mo suggests that the observed DNA damage consists mainly of single-strand breaks, a sensitive biomarker induced by reactive oxygen species and reversible over time (*29*). Indeed, cancer, chronic inflammation, and oxidative stress are closely related, and numerous agents, such as ionizing radiation, have been proven to interfere with redox cell signaling pathways (*30*). Since several studies have shown that unstable DNA damage can also manifest several cellular generations after radiation exposure, generating what is called delayed damage, this study also assessed the unstable damage at 3 mo after therapy, by CometAssay, but we found no significant difference between the 2 groups.

Stable aberrations, by contrast, are a marker of radiation exposure, and the results of the M-FISH analysis showed a statistically significant increase in the frequency of chromosome breaks between the basal sample and the 3-mo sample only in HYPO patients, suggesting that in these patients, not prepared with rhTSH, genomic instability occurs after treatment and persists with time. These data could be partially explained by the reduced renal clearance of ^131^I (due to a reduced glomerular filtration rate) and higher retained total-body activity (due to the hypothyroidism induced in HYPO patients by withdrawal of levothyroxine), but genetic and metabolic factors could also play a role.

Use of rhTSH, by maintaining euthyroidism and a normal renal clearance (epi-GFR and creatinine values), reduced by 27% the radiation exposure rate over a period of 120 h, an indirect measurement of the retained body activity, and reduced the genomic instability.

SNPs represent different variants of the same gene present in the population. In each individual, the type and combination of polymorphisms of genes involved in DNA repair, and for enzymes acting as free oxygen radical scavengers, influenced the amount of DNA damage observed. Thus, analysis of gene polymorphism allowed us to identify a subgroup of HYPO and rhTSH patients more susceptible to chromosome damage induced by ^131^I because of mutations in enzymes deputed to DNA repair (DNA-1) or enzymes involved in scavenging of free oxygen radicals (DNA-2). It was interesting to note that among these patients, the HYPO group showed a greater increase in chromosome damage after ^131^I than did the rhTSH group. Indeed, polymorphism of the gene regulating the redox status has been involved in several other malignancies, mainly breast cancer (*31*).

Overall, in HYPO patients the number of chromosome breaks at 3 mo was associated with the level of breaks at baseline, residual body activity at 48 h, and levels of anti-ROMs (Table 2). As far as the anti-ROMs are concerned, we found a significant positive association with anti-ROMsF at 1 wk and a negative association with anti-ROMsS at 1 wk. An explanation for this finding could be that a high level of dROMs (as observed in these patients) induces an increase in anti-ROMs and that, among these, anti-ROMsF rises quickly and is positively associated with an increase in chromosome damage, whereas anti-ROMsS may require more time to rise and is negatively associated with an increase in chromosome damage.

Patients prepared with rhTSH showed no significant increase in either breaks or total exchanges after ^131^I, suggesting that these patients have less radiation-induced chromosome damage, even in the presence of mutations in several enzymes. Interestingly, the number of chromosome breaks at 3 mo in rhTSH patients was positively associated with polymorphisms of genes for DNA-repairing enzymes (DNA-2 group).

These data confirm previously published findings that ^131^I therapy can induce stable DNA damage, but for the first time (to our knowledge) we were able to demonstrate that rhTSH may significantly reduce this damage, particularly in patients with unfavorable polymorphisms of genes involved in DNA repair. Whether chromosome damage in peripheral lymphocytes relates to an increased risk of secondary malignancies remains a matter of debate. In addition, data obtained by measurement of d-ROMs and anti-ROMs demonstrate a crucial role of oxidative stress. In this regard, patients with high levels of d-ROMs and low levels of anti-ROMs, or with an unfavorable genotype for DNA-repairing enzymes or for free oxygen radical scavengers, might be more susceptible to radiation-induced DNA damage.

## CONCLUSION

Administered activity, DNA polymorphisms, glomerular filtration rate, and oxidant/antioxidant homeostasis are all parameters that may influence DNA damage in patients treated with ^131^I. Our study highlighted the importance of rhTSH in preventing radiation-induced stable chromosome damage, even if an unfavorable genetic background is present. Even if stable DNA damage is considered particularly important for involvement in tumor cell clonal evolution, our study showed no evidence that stable DNA damage has clinical consequences or induces secondary tumors. To evaluate these specific endpoints, larger longitudinal studies are warranted.

## Notes

KEY POINTS **QUESTION:** Is there transient DNA damage after ^131^I ablation therapy, is there detectable permanent ^131^I-induced chromosome damage, and to what extent is permanent chromosome damage due to ^131^I dose or to oxidative stress in the patient and gene polymorphism? **PERTINENT FINDINGS:** DNA damage after ^131^I ablation therapy occurred in most patients treated with 1,850 MBq and disappeared after 3 mo. Some chromosome breaks and exchanges occurred after ^131^I therapy, particularly in HYPO patients, possibly because of higher retained activity or high oxidative stress. Most patients had a reduction in anti–free oxygen radical scavengers and an increase in free oxygen radicals. An unfavorable genotype exposed patients to higher radiation-induced damage. When patients were matched for severity of gene mutations, hypothyroid patients had higher levels of chromosome damage. **IMPLICATIONS FOR PATIENT CARE:** Transient chromosome damage is not relevant in patients treated with 1,850 MBq of ^131^I. Candidates for ^131^I ablation therapy should be prepared with rhTSH, avoiding discomfort and symptoms of hypothyroidism and chromosome damage. Genes that control oxidative stress might influence induction of DNA damage in patients treated with ^131^I.
